# Pioglitazone as Add-On to Metformin and Dapagliflozin Yields Significant Enhancements in Glycemic Control in Poorly Controlled Type 2 Diabetes: A Meta-Analysis of Randomized Controlled Trials

**DOI:** 10.7759/cureus.92794

**Published:** 2025-09-20

**Authors:** Sara Sabbagh, Ahmed Hegazy, Ahmed Adel, Abdullah Ali, Mohamed Alquddosy, Sara Khalid, Abdallah M Ibrahim, Ahmed Mohamed, Abdelrahman Mostafa, Ahmed Hassan, Ola Mohamed, Rawan Mesbah, Osama Osman, Mohamed Hamouda Elkasaby

**Affiliations:** 1 Faculty of Medicine, Damascus University, Damascus, SYR; 2 Acute Medicine, Medway NHS Foundation Trust, Gillingham, GBR; 3 Faculty of Medicine, Zagazig University, Zagazig, EGY; 4 Faqous Faculty of Medicine, Zagazig University, Faqous, EGY; 5 Faculty of Medicine, Mansoura University, Mansoura, EGY; 6 Faculty of Medicine, Helwan University, Cairo, EGY; 7 Faculty of Medicine, Alexandria University, Alexandria, EGY; 8 Faculty of Medicine, Al-Azhar University, Cairo, EGY; 9 Faculty of Medicine, October 6th University, 6th of October City, EGY; 10 Faculty of Medicine, Mari State University, Yoshkar-ola, RUS

**Keywords:** dapagliflozin, metformin, pioglitazone, triple oral therapy, type 2 diabetes mellitus

## Abstract

This systematic review and meta-analysis aimed to evaluate the efficacy and safety of pioglitazone as an add-on therapy to dual treatment with metformin and dapagliflozin in adults with type 2 diabetes mellitus (T2DM) inadequately controlled on this regimen. We conducted a systematic review and meta-analysis of randomized controlled trials (RCTs) comparing pioglitazone (15 or 30 mg) versus placebo as an add-on to metformin (1,000 mg/day) and dapagliflozin (10 mg/day). Four databases (PubMed, Scopus, Cochrane CENTRAL, and Web of Science) were searched through December 2024. Primary outcomes included changes in hemoglobin A1c (HbA1c), the proportion of patients achieving a therapeutic glycemic response, and fasting plasma glucose (FPG). Secondary outcomes were insulin resistance, β-cell function, lipid parameters, body weight, blood pressure, and adverse events. Mean difference (MD) and standard deviation (SD) were used to describe the continuous variables. For categorical variables, we used the risk ratio (RR) and 95% confidence interval (CI). We included three RCTs, comprising a total of 856 patients: 363 received pioglitazone 15 mg, 124 received the 30 mg dose, and 369 received a placebo. Pioglitazone 15 mg significantly reduced HbA1c (MD = -0.42 percentage point, 95% CI = -0.51 to -0.33; p < 0.00001) and FPG (MD = -12.41 mg/dL). The 30 mg dose yielded greater reductions in HbA1c (MD = -0.84 percentage point) and FPG (MD = -21.49 mg/dL). It also improved homeostatic model assessment of insulin resistance, high-density lipoprotein cholesterol, and triglycerides, but increased body weight. No significant differences in serious adverse events or hypoglycemia were observed. Most outcomes had moderate to high certainty. Adding pioglitazone to metformin and dapagliflozin significantly enhances glycemic control and improves metabolic parameters with an acceptable safety profile, supporting its role as an effective triple oral therapy in T2DM.

## Introduction and background

Type 2 diabetes mellitus (T2DM) is a chronic metabolic disorder characterized by insulin resistance and progressive β-cell dysfunction, leading to impaired glucose homeostasis [1]. It affects about 462 million adults worldwide, representing 6.28% of the global population, and its prevalence is rising rapidly, with major complications including cardiovascular disease, nephropathy, neuropathy, and retinopathy [2-4]. Therefore, effective glycemic control is essential to reduce long-term morbidity and mortality [4].

The cornerstone of T2DM management is a multifaceted approach that includes lifestyle modifications, such as dietary changes, regular physical activity, and weight management [5]. However, for many individuals, lifestyle interventions alone are insufficient to achieve adequate glycemic control. Therefore, pharmacological therapy plays a crucial role in the management of most individuals with T2DM [6].

Metformin remains the first-line pharmacological agent for most T2DM patients, primarily reducing hepatic glucose production and enhancing peripheral insulin sensitivity [7]. However, many require adjunctive therapies for optimal glycemic control [8]. Sodium-glucose cotransporter-2 (SGLT2) inhibitors, such as dapagliflozin, lower plasma glucose levels by inhibiting renal glucose reabsorption, thereby promoting urinary glucose excretion [9]. Beyond glycemic control, they have also demonstrated robust cardiovascular and renal benefits in large clinical trials [9].

Despite these benefits, some patients may still experience inadequate glycemic control [10]. In such cases, pioglitazone, a thiazolidinedione and peroxisome proliferator-activated receptor-γ (PPAR-γ) agonist, may offer therapeutic benefit by improving insulin sensitivity in skeletal muscle and adipose tissue, reducing hepatic glucose output, enhancing glycemic control, and favorably influencing lipid profiles [11-13].

However, evidence regarding the efficacy and safety of adding pioglitazone to patients already receiving metformin and dapagliflozin is scarce, and this combination has not been systematically evaluated. This review aims to address this gap by synthesizing available evidence on this triple therapy regimen in poorly controlled T2DM.

## Review

Methodology

We followed the Cochrane Handbook of Systematic Reviews of Interventions as guidance [14]. When reporting this systematic review and meta-analysis, we adhered to the updated Preferred Reporting Items for Systematic Reviews and Meta-Analyses (PRISMA) guidelines [15]. The protocol was registered on the International Prospective Register of Systematic Reviews (PROSPERO registration number: CRD42024598310) [16].

Eligibility Criteria

This meta-analysis included randomized controlled trials (RCTs) that assessed the efficacy and safety of pioglitazone as an add-on therapy in adults with type 2 diabetes, following the PICOS framework. Population: adults with type 2 diabetes, a body mass index (BMI) below 45 kg/m², and baseline glycated hemoglobin (HbA1c) levels between 7.0% and 11.0%. Intervention: pioglitazone (15 or 30 mg/day) added to a background therapy of metformin (1,000 mg/day) and dapagliflozin. Comparator: placebo combined with metformin (1,000 mg/day) and dapagliflozin (10 mg/day). The primary outcomes were the mean change in HbA1c from baseline after 24 weeks, the proportion of patients achieving a therapeutic glycemic response, defined as HbA1c <7.0% and <6.5%, at 24 weeks, in addition to the changes in fasting plasma glucose (FPG). Secondary outcomes included homeostatic model assessment of insulin resistance (HOMA-IR), homeostatic model assessment of β-cell function (HOMA-β), lipid profile parameters, including total cholesterol, low-density lipoprotein cholesterol (LDL-C), high-density lipoprotein cholesterol (HDL-C), and triglycerides, as well as body weight, systolic and diastolic blood pressure, and safety outcomes such as treatment-emergent adverse events (TEAEs) and serious adverse events (SAEs). Studies were excluded if they were animal studies, case series, case reports, theses, conference abstracts, or if the full text was not available.

Search Strategy

A comprehensive literature search was performed on the PubMed, Scopus, Web of Science, and Cochrane CENTRAL databases from inception until December 10th, 2024. We used a combination of keywords and MeSH (Medical Subject Headings) terms for a sensitive search strategy: “(Pioglitazone OR AD4833 OR U72107A OR Pioglitazone Hydrochloride OR Actos) AND (Dapagliflozin OR BMS512148 OR Farxiga OR Forxiga) AND (Metformin OR Dimethylbiguanidine OR Glucophage OR Dimethylguanylguanidine OR Metformin Hydrochloride) AND (Type 2 diabetes OR Type 2 DM).” Further details of each database search strategy are shown in Appendix 1.

Study Selection

We used Rayyan to remove duplicates [17]. Two authors independently conducted a two-step screening process (AA and MO): first, reviewing titles and abstracts, followed by full-text assessment against the previously structured eligibility criteria. We also conducted manual citation analysis on all references of the included studies to ensure comprehensive coverage of relevant studies.

Data Extraction

Two reviewers (AA and AH) independently extracted data from each included study using a standardized data extraction form. Any discrepancies were resolved through discussion or consultation with a third reviewer (AI). Extracted data included study characteristics (author, year of publication, study design, setting), participant characteristics (sample size, mean age, sex distribution, baseline BMI, duration of diabetes, and baseline HbA1c), details of interventions (dose and duration of pioglitazone, background therapy with metformin and dapagliflozin), and outcomes of interest.

Primary outcomes included changes in HbA1c at 12 and 24 weeks and the proportion of participants achieving HbA1c targets (<7% and <6.5%). Secondary outcomes included changes in FPG, HOMA-IR, HOMA-β, lipid profile parameters (total cholesterol, LDL-C, HDL-C, triglycerides), body weight, systolic and diastolic blood pressure, and safety outcomes (TEAEs and SAEs). All outcomes were extracted at 24 weeks of follow-up, except as mentioned.

Management Risk of Bias Assessment

We used the Cochrane risk-of-bias tool (ROB 2) for the included RCTs [18], which has the following five domains: (1) randomization process, (2) deviation from intended intervention, (3) missing outcome data, (4) measurement of the outcome, and (5) selection of the reported outcome. At every stage of each domain, three authors (SS, AE, and SE) had to decide (yes, no, probably yes, probably no, or no information).

Statistical Analysis

We conducted the statistical analysis using Review Manager (RevMan) version 5.4 (Cochrane Collaboration, 2020). For continuous outcomes, we calculated the mean difference (MD) with 95% confidence intervals (CIs), while for categorical outcomes, we used risk ratios (RRs) with 95% CIs. A p-value of less than 0.05 was considered statistically significant. Data from individual studies were pooled using the inverse-variance method for continuous outcomes and the Mantel-Haenszel method for categorical outcomes.

Heterogeneity among studies was assessed through visual inspection of forest plots and quantified using the chi-square (χ²) test and the I² statistic. The chi-square test indicates the presence of significant heterogeneity (with p < 0.10 considered significant), while I² describes the percentage of total variation across studies due to heterogeneity rather than chance. I² values were interpreted as follows: 0-20% may not be important, 30-60% may indicate moderate heterogeneity, and 50-90% may represent substantial heterogeneity. In cases of significant heterogeneity, a random-effects model was applied instead of a fixed-effect model, and sensitivity analyses were conducted by sequentially excluding one study at a time.

In Cho et al (2024), both 15 mg and 30 mg pioglitazone doses were reported. We, therefore, conducted a subgroup analysis by dose (15 mg vs. 30 mg), and separate pooled estimates were calculated accordingly. A chi-square test for subgroup differences was performed. Meta-regression was not conducted due to the limited number of included studies. According to Egger et al. [19], assessment is not reliable for fewer than 10 studies. Therefore, we could not assess the existence of publication bias in the present study.

Certainty of the Evidence

We assessed the certainty of evidence using the Grading of Recommendations Assessment, Development and Evaluation (GRADE) approach [20]. The overall results are presented in Table 1 as a summary of findings (SoF), and the detailed GRADE evidence profiles are provided in Appendix 2. The GRADE framework considers the following five domains: risk of bias, inconsistency, indirectness, imprecision, and publication bias, to rate the certainty of evidence as high, moderate, low, or very low. High certainty indicates strong confidence that the true effect is close to the estimated effect. Moderate certainty suggests the true effect is likely close but may differ substantially. Low certainty reflects limited confidence, with a considerable chance that the true effect is substantially different. Very low certainty indicates very little confidence in the estimate, implying the true effect is likely to be substantially different.

**Table 1:** Summary of findings grading of recommendations, assessment, development and evaluation (GRADE). HbA1c: hemoglobin A1c; FPG: fasting plasma glucose; HOMA-IR: homeostatic model assessment of insulin resistance; HOMA-B: homeostatic model assessment of beta-cell function; HDL: high-density lipoprotein; LDL: low-density lipoprotein; SBP: systolic blood pressure; DBP: diastolic blood pressure; RR: relative risk; MD: mean difference; CI: confidence interval

Outcomes	Follow-up	Number of RCTs	Number of patients	Mean treatment effect (95% CI)	Statistical heterogeneity	GRADE quality rating
HbA1c	Range 1 week to 24 weeks	3	732	MD 0.42 lower (0.51 lower to 0.33 lower)	I² = 0%	⨁⨁⨁⨁ High
HbA1c	Range 1 week to 12 weeks	3	732	MD 0.33 % lower (0.41 lower to 0.25 lower)	I² = 0%	⨁⨁⨁⨁ High
HbA1c <7%	Range 1 week to 24 weeks	3	718	RR 2.08 (1.65 to 2.62)	I² = 0%	⨁⨁⨁⨁ High
HbA1c <6.5%	Range 1 week to 24 weeks	3	718	RR 2.46 (1.54 to 3.93)	I² = 0%	⨁⨁⨁⨁ High
FPG	Range 1 week to 24 weeks	3	732	MD 12.41 mg/dL lower (15.66 lower to 9.15 lower)	I² = 0%	⨁⨁⨁⨁ High
FPG	Range 1 week to 12 weeks	3	732	MD 10.6 % lower (14.11 lower to 7.1 lower)	I² = 0%	⨁⨁⨁⨁ High
HOMA-IR	Range 1 week to 24 weeks	3	732	MD 0.79 % lower (1.06 lower to 0.53 lower)	I² = 32%	⨁⨁⨁⨁ High
HOMA-B	Range 1 week to 24 weeks	2	492	MD 1.08 % higher (5.84 lower to 8 higher)	I² = 51%	⨁⨁⨁◯ Moderate
Total cholesterol	Range 1 week to 24 weeks	3	732	MD 1.45 mg/dL higher (2.43 lower to 5.33 higher)	I² = 0%	⨁⨁⨁◯ Moderate
HDL	Range 1 week to 24 weeks	3	732	MD 2.93 mg/dL higher (1.61 higher to 4.25 higher)	I² = 64%	⨁⨁⨁◯ Moderate
LDL	Range 1 week to 24 weeks	3	732	MD 1.18 mg/dL lower (4.58 lower to 2.22 higher)	I² = 64%	⨁⨁⨁◯ Moderate
Triglyceride	Range 1 week to 24 weeks	3	732	MD 13.01 mg/dL lower (23.78 lower to 2.24 lower)	I² = 0%	⨁⨁⨁◯ Moderate
Body weight	Range 1 week to 24 weeks	3	732	MD 2.23 higher (1.88 higher to 2.57 higher)	I² = 23%	⨁⨁⨁⨁ High
SBP	Range 1 week to 24 weeks	3	732	MD 0.85 mmHg lower (2.56 lower to 0.86 higher)	I² = 59%	⨁⨁◯◯ Low
DBP	Range 1 week to 24 weeks	3	732	MD 1.48 mmHg lower (2.67 lower to 0.29 lower)	I² = 72%	⨁⨁◯◯ Low
Treatment-emergent adverse events	Range 1 week to 24 weeks	3	757	RR 1.04 (0.82 to 1.32)	I² = 0%	⨁⨁⨁◯ Moderate
Serious adverse events	Range 1 week to 24 weeks	2	510	RR 1.8 (0.61 to 5.29)	I² = 0%	⨁⨁⨁◯ Moderate

Results

Study Selection and Characteristics

**Figure 1:**
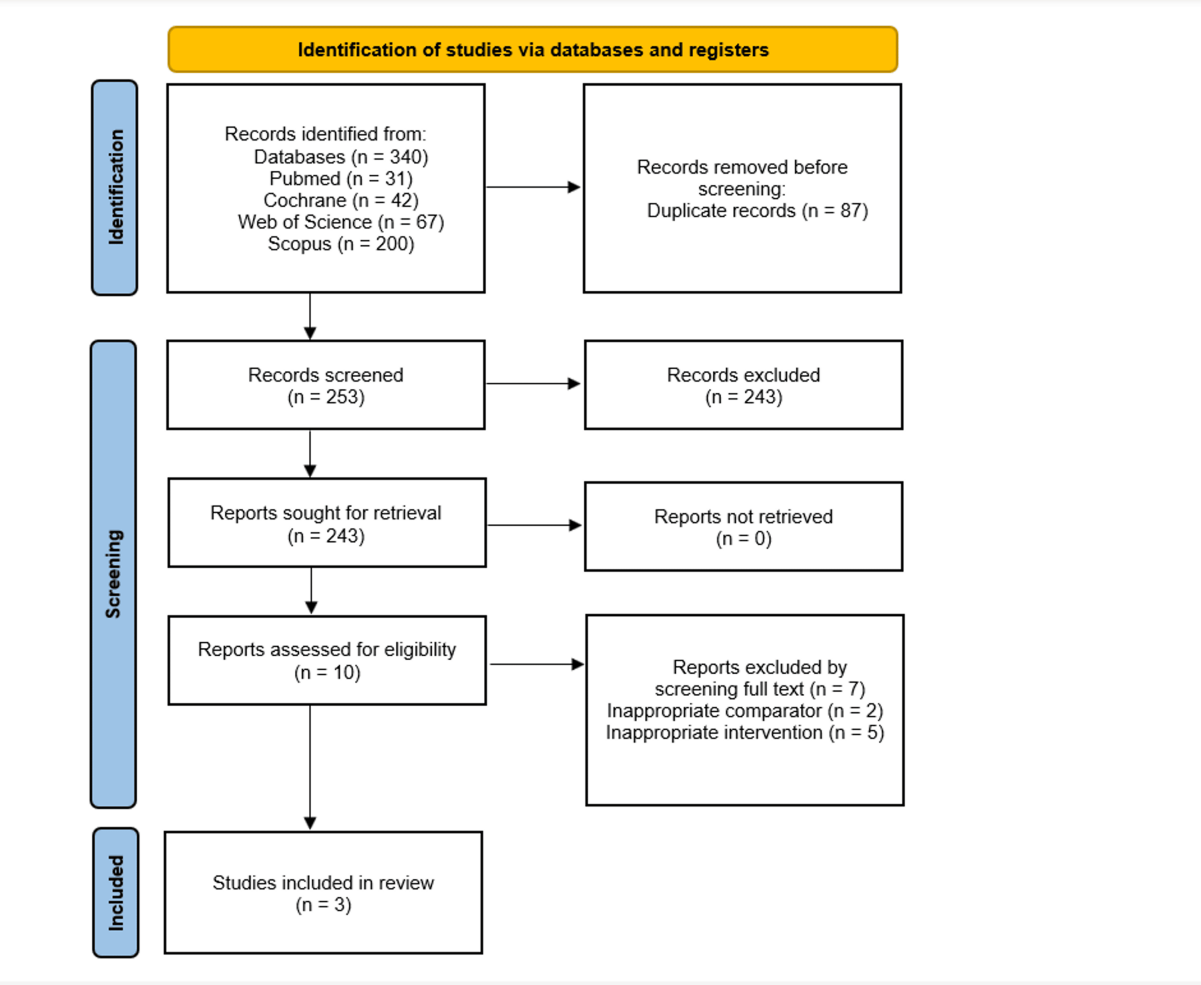
Preferred Reporting Items for Systematic Reviews and Meta-Analyses (PRISMA) flow diagram illustrating the study selection process for the meta-analysis.

A comprehensive search of electronic databases initially identified 340 studies. After removing duplicates, 253 articles remained for title and abstract screening, of which only 10 were deemed eligible for full-text review. Out of these, two studies were excluded due to inappropriate comparators, while the interventions of five other articles did not match our standards. Ultimately, three RCTs [10,21,22] met the inclusion criteria for the meta-analysis, comprising a total of 856 patients. The study selection process is presented in Figure 1.

Characteristics of Included Studies

We included three RCTs, comprising a total of 856 patients: 363 received pioglitazone 15 mg, 124 received the 30 mg dose, and 369 received a placebo. All studies included in the analysis were conducted in Korea and published in 2024. The treatment period of these studies lasted for 24 weeks. All studies used a 15 mg dose of pioglitazone, while Cho et al. (2024) [21] used a 15 mg dose of pioglitazone and an additional dose of 30 mg. The summary characteristics of the included studies are summarized in Table 2.

**Table 2:** Summary characteristics of the included studies. RCT: randomized controlled trial

Study ID	Study design	Location	Sample size	Intervention	Comparator	Follow-up duration	Trial registration number
Cho et al. (2024) [21]	RCT	South Korea	374	Pioglitazone 15 mg daily/30 mg daily	Placebo	24 weeks	NCT04885712
Heo et al. (2024) [22]	RCT	South Korea	262	Pioglitazone 15 mg daily	Placebo	24 weeks	NCT05101135
Lim et al. (2024) [10]	RCT	South Korea	249	Pioglitazone 15 mg daily	Placebo	24 weeks	NCT05226897

Baseline characteristics of participants across the included studies are presented in Table 3. The mean age ranged from 55.0 to 58.2 years, with the proportion of male participants ranging between 48.0% and 61.9%. Duration of diabetes varied from 8.2 to 10.2 years. Baseline HbA1c levels ranged from 7.62% to 8.0%, and FPG values ranged from 131.6 to 144.6 mg/dL. Participants had similar BMI values, ranging from 25.5 to 27.0 kg/m². Systolic blood pressure (SBP) and diastolic blood pressure (DBP) were consistent across groups, as were lipid parameters, including total cholesterol, triglycerides, HDL-C, and LDL-C. HOMA-IR and HOMA-β values were also comparable between treatment and placebo arms.

**Table 3:** Baseline characteristics of the included studies. HbA1c: glycated hemoglobin; FPG: fasting plasma glucose; BMI: body mass index; SBP: systolic blood pressure; DBP: diastolic blood pressure; HDL-C: high-density lipoprotein cholesterol; LDL-C: low-density lipoprotein cholesterol; HOMA-IR: homeostatic model assessment of insulin resistance; HOMA-β: homeostatic model assessment of beta-cell function

Study	Treatment group	Number of participants	Age, years	Gender, Male	Duration of diabetes, years	HbA1c, mg/dL	FPG, mg/dL	Weight, Kg	BMI, kg/m2	SBP, mm Hg	DBP, mm Hg	Total cholesterol, mg/dL	Triglyceride, mg/dL	HDL-C, mg/dL	LDL-C, mg/dL	HOMA-IR	HOMA-𝛽, percentage points
Cho et al. (2024) [21]	Pioglitazone (30 mg)	124	55.2 (±11.8)	69 (55.7%)	8.3 (±5.6)	8.0 (±0.7)	139.7 (±30.5)	71.4 (±13.6)	26.3 (±3.3)	123.6 (±12.1)	75.1 (±9.4)	152.8 (±37.4)	122.0 (49.0–396.0)	49.1 (±11.9)	83.6 (±32.2)	2.8 (±2.1)	39.8 (±27.8)
	Pioglitazone (±15mg)	118	56.8 (±11.0)	73 (61.9%)	9.5 (±6.0)	7.9 (±0.7)	137.5 (±24.2)	71.7 (±15.4)	26.0 (±4.0)	126.0 (±13.4)	75.9 (±9.4)	152.3 (±33.2)	133.5 (47.0–1,448.0)	47.2 (±11.3)	82.4 (±29.9)	3.3 (±4.1)	52.1 (±98.7)
	Placebo	124	55.0 (±10.3)	66 (53.2%)	8.2 (±5.6)	7.9 (±0.7)	140.1 (±26.4)	73.2 (±15.2)	27.0 (±4.2)	127.32 (±11.57)	76.41 (±9.02)	146.48 (±32.43)	130.76 (±76.91)	50.28 (±9.32)	79.42 (±27.72)	2.86 (±1.90)	47.04 (±32.10)
Heo et al. (2024) [22]	Pioglitazone (±15mg)	125	57.6 (±10.0)	61 (48.8%)	9.6 (±5.7)	7.62 (±0.55)	133.80 (±23.42)	71.0 (±11.9)	26.3 (±3.4)	127.37 (±11.92)	77.34 (±8.51)	151.82 (±33.16)	136.40 (±70.41)	49.43 (±11.70)	83.78 (±28.61)	2.56 (±2.04)	42.00 (±28.24)
	Placebo	125	56.9 (±10.3)	60 (48.0%)	8.6 (±6.3)	7.76 (±0.77)	131.60 (±20.49)	70.1 (±11.4)	26.4 (±3.7)	124.8 (±13.8)	74.3 (±9.5)	151.8 (±37.1)	133.0 (±72.7)	50.5 (±13.8)	83.6 (±33.8)	3.0 (±3.1)	-
Lim et al. (2024) [10]	Pioglitazone (±15mg)	120	57.7 (±10.0)	70 (56.5%)	10.2 (±6.5)	7.8 (±0.7)	142.9 (±22.3)	68.6 (±11.4)	25.8 (±3.4)	125.2 (±14.3)	74.3 (±9.5)	151.3 (±33.5)	147.8 (±104.1)	49.9 (±13.8)	81.4 (±30.2)	2.4 (±1.5)	-
	Placebo	120	58.2 (±10.1)	64 (51.2%)	10.1 (±5.9)	7.8 (±0.8)	144.6 (±27.8)	69.1 (±11.1)	25.5 (±3.0)	-	-	-	-	-	-	-	-

Quality of Included Studies

**Figure 2:**
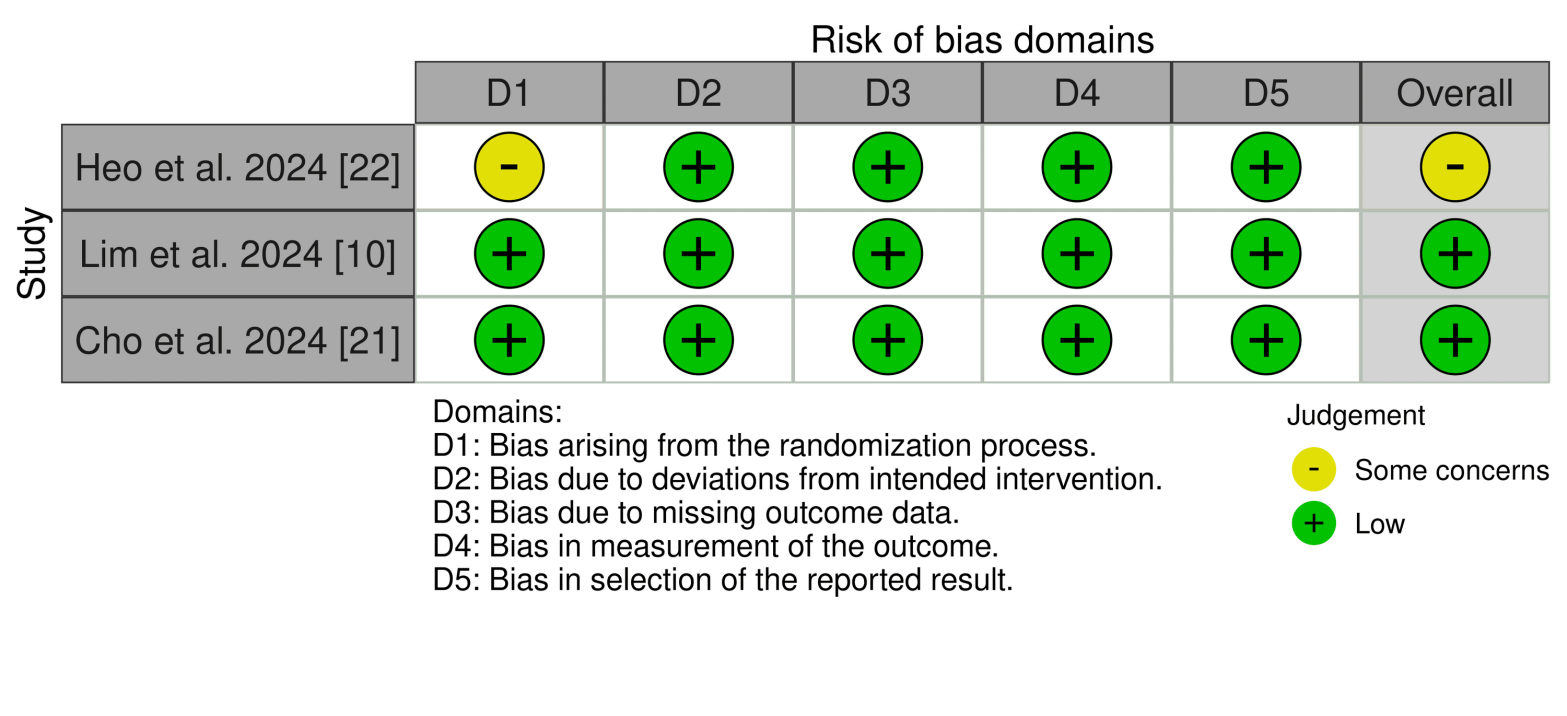
Summary of risk of bias judgments across all domains for each included randomized controlled trial, assessed using the Cochrane Risk of Bias 2 tool.

**Figure 3:**
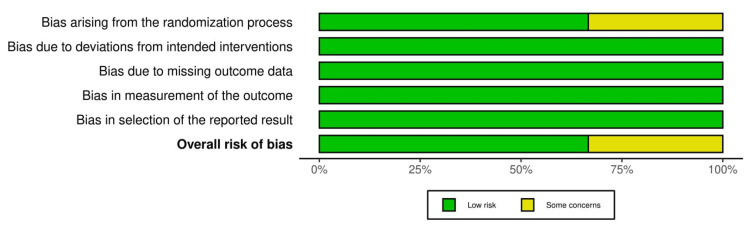
Overall risk of bias assessment for the included studies.

All three studies (Heo et al. (2024), Lim et al. (2024), and Cho et al. (2024)) were judged to be at low risk of bias in all domains; only Heo et al. (2024) was rated as having “some concerns” in the randomization process, due to limited information on the allocation concealment method [10,21,22] (Figures 2, 3). The detailed authors’ judgments and supporting rationales are provided in Appendices 3-8.

Primary Clinical Outcomes

*Glycosylated Hemogl*obin

**Figure 4:**
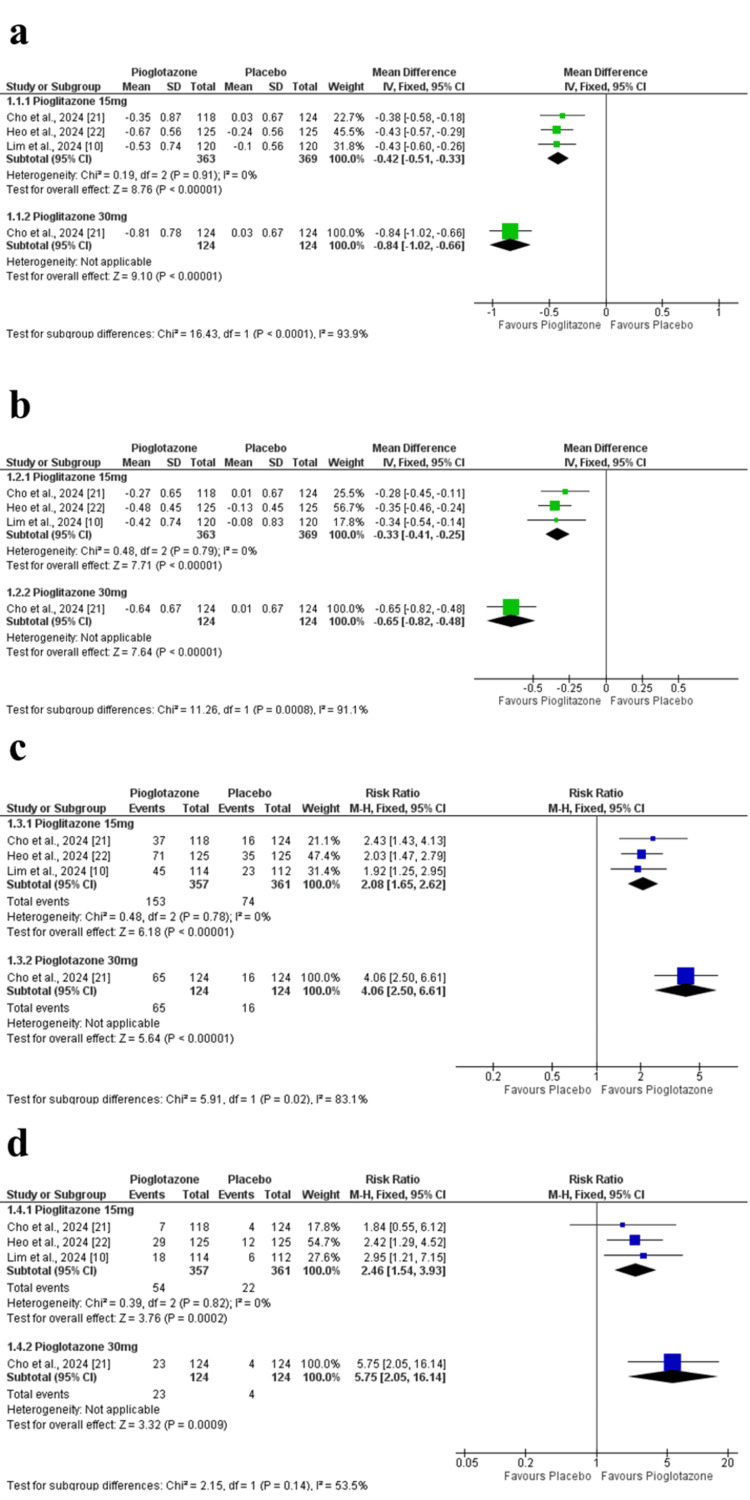
Forest plots of HbA1c-related outcomes. (a) Change in HbA1c at 24 weeks. (b) Change in HbA1c at 12 weeks. (c) Proportion of participants achieving HbA1c <7%. (d) Proportion of participants achieving HbA1c <6.5%. HbA1c: glycated hemoglobin

Change in HbA1c at 24 weeks: The pooled analysis showed a significant reduction in HbA1c at 24 weeks in the pioglitazone 15 mg group compared to placebo (MD = -0.42 percentage point; 95% CI = -0.51 to -0.33; p < 0.00001), with no observed heterogeneity among studies (p = 0.91, I² = 0%). Additionally, in Cho et al. (2024), the higher 30 mg dose of pioglitazone demonstrated a more pronounced effect (MD = -0.84 percentage point; 95% CI = -1.02 to -0.66; p < 0.00001) (Figure 4a). The certainty of evidence was rated as high.

Change in HbA1c at 12 weeks: At 12 weeks, pioglitazone 15 mg was also superior to placebo (MD = -0.33 percentage point; 95% CI = -0.41 to -0.25; p < 0.00001), with homogenous study results (p = 0.79, I² = 0%). In Cho et al. (2024), the 30 mg dose yielded a greater HbA1c reduction (MD = -0.65 percentage point; 95% CI = -0.82 to -0.48; p < 0.00001) (Figure 4b). The certainty of evidence was rated as high.

Achievement of HbA1c <7%: More patients in the pioglitazone 15 mg group achieved HbA1c <7% compared to placebo (RR = 2.08; 95% CI = 1.65 to 2.62; p < 0.00001), with no heterogeneity detected (p = 0.78, I² = 0%). Similarly, Cho et al. (2024) reported a stronger effect with the 30 mg dose (RR = 4.06; 95% CI = 2.50 to 6.61; p < 0.00001) (Figure 4c). The certainty of evidence was rated as high.

Achievement of HbA1c <6.5%: Pioglitazone 15 mg also resulted in a higher proportion of patients reaching HbA1c <6.5% compared to placebo (RR = 2.46; 95% CI = 1.54 to 3.93; p = 0.0002), with low heterogeneity (p = 0.82, I² = 0%). The 30 mg dose in Cho et al. (2024) showed a greater effect (RR = 5.75; 95% CI = 2.05 to 16.14; p = 0.0009) (Figure 4d). The certainty of evidence was rated as high.

Fasting Plasma Glucose

**Figure 5:**
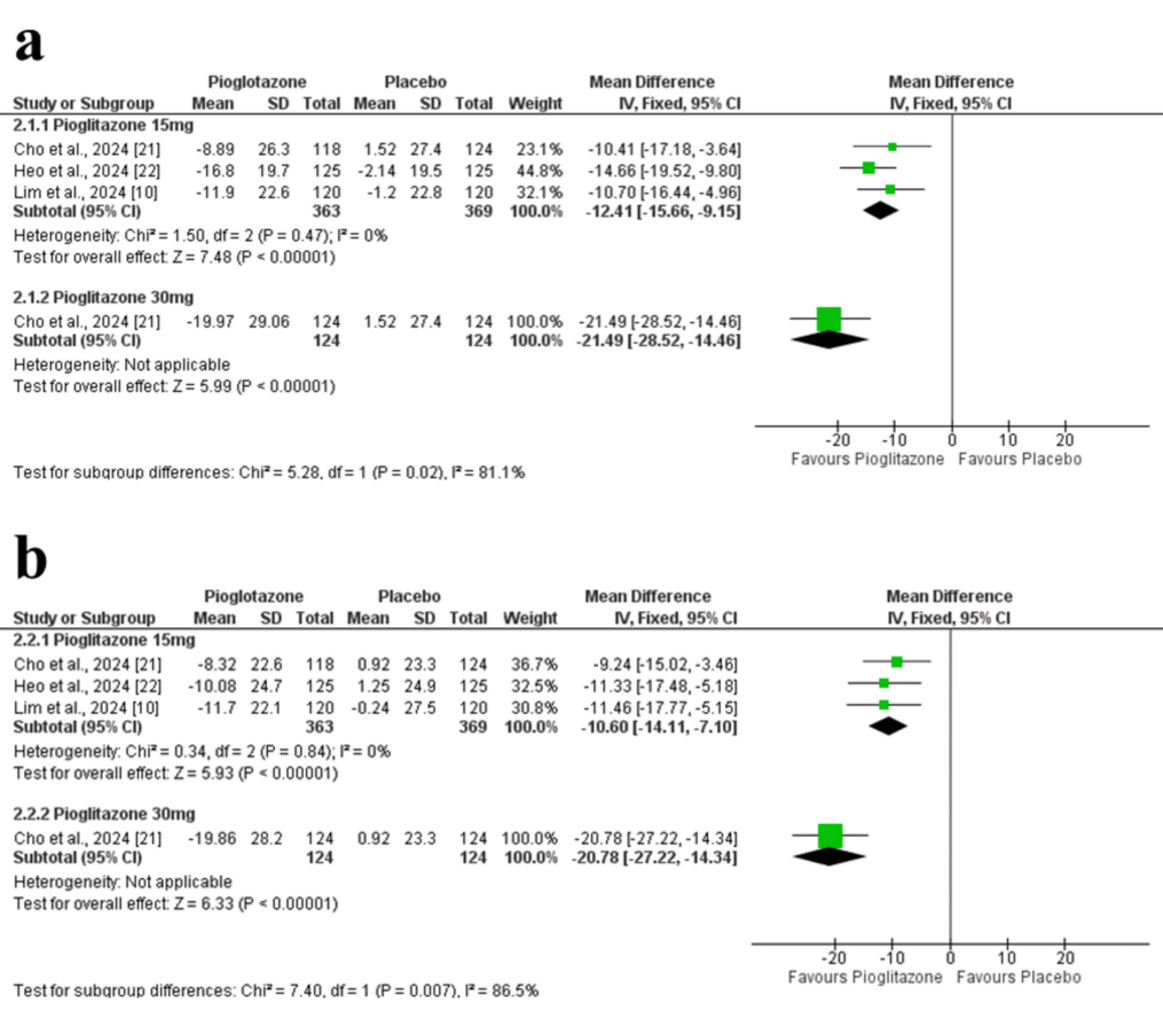
Forest plots of FPG outcomes. (a) Change in FPG at 24 weeks. (b) Change in FPG at 12 weeks. FPG: fasting plasma glucose

Change in FPG at 24 weeks: Pioglitazone 15 mg significantly reduced FPG levels compared to placebo (MD = -12.41 mg/dL; 95% CI = -15.66 to -9.15; p < 0.00001), with no heterogeneity (p = 0.47, I² = 0%). In Cho et al. (2024), the 30 mg dose had a stronger effect (MD = -21.49 mg/dL; 95% CI = -28.52 to -14.46; p < 0.00001) (Figure 5a). The certainty of evidence was rated as high.

Change in FPG at 12 weeks: At 12 weeks, pioglitazone 15 mg also led to a significant reduction in FPG (MD = -10.60 mg/dL; 95% CI = -14.11 to -7.10; p < 0.00001). The 30 mg dose in Cho et al. (2024) showed greater efficacy (MD = -20.78 mg/dL; 95% CI = -27.22 to -14.34; p < 0.00001) (Figure 5b). The certainty of evidence was rated as high.

Secondary Clinical Outcomes

**Figure 6:**
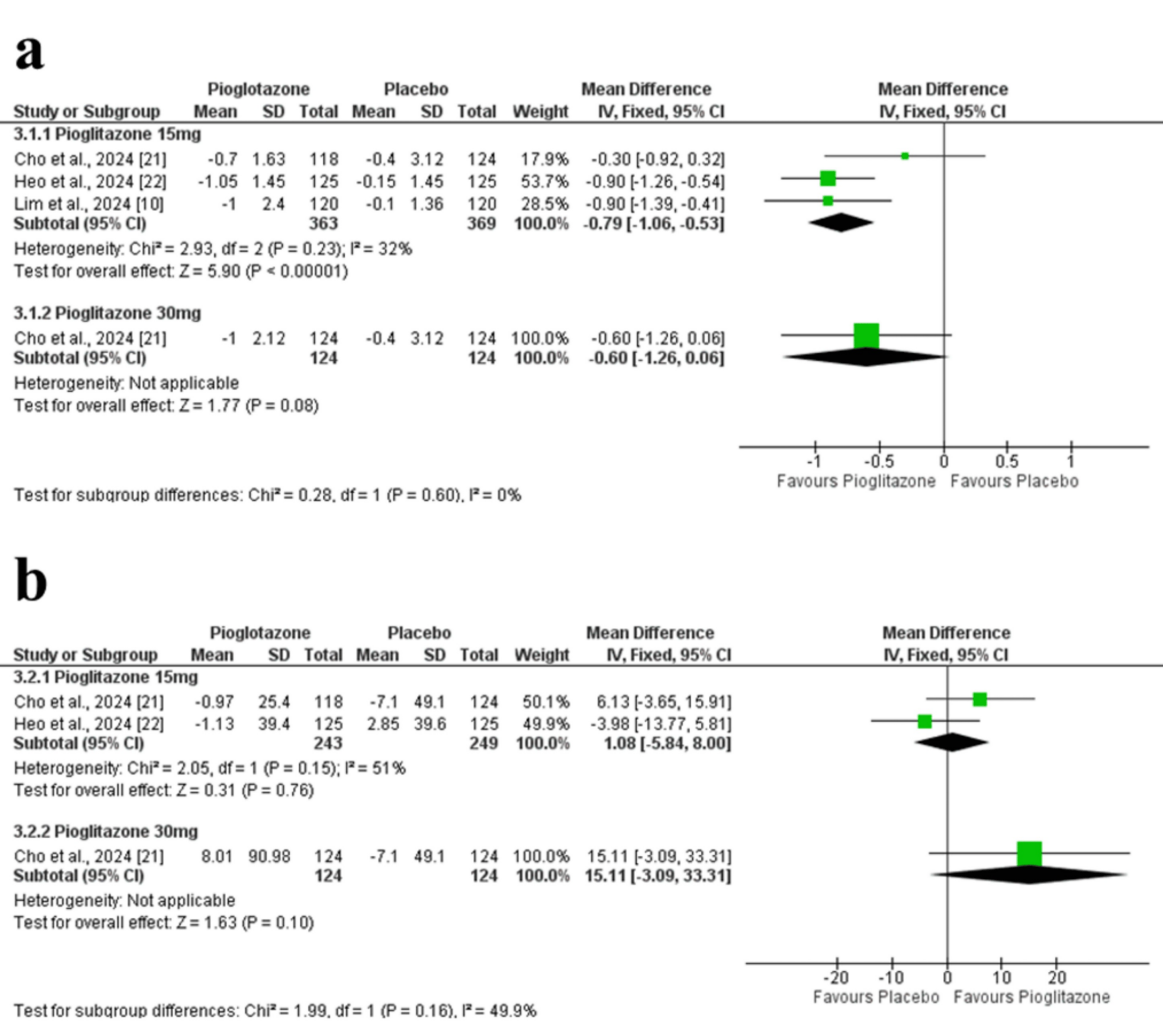
Forest plots of insulin sensitivity and β-cell function at 24 weeks. (a) Change in HOMA-IR. (b) Change in HOMA-β. HOMA-IR: homeostatic model assessment of insulin resistance; HOMA-B: homeostatic model assessment of beta-cell function

Homeostatic model assessment of insulin resistance: Pioglitazone 15 mg significantly improved insulin resistance (MD = -0.79; 95% CI = -1.06 to -0.53; p < 0.00001) with mild heterogeneity (p = 0.23, I² = 32%). The 30 mg dose in Cho et al. (2024) did not show a significant difference (MD = -0.60; 95% CI = -1.26 to 0.06; p = 0.08) (Figure 6a). The certainty of evidence was rated as high.

Homeostatic model assessment of β-cell function: Two studies reported on HOMA-β, showing no significant difference between pioglitazone 15 mg and placebo (MD = 1.08%; 95% CI = -5.84 to 8.00; p = 0.76; I² = 51%). The 30 mg dose in Cho et al. (2024) also showed no significant change (MD = 15.11%; 95% CI = -3.09 to 33.31; p = 0.10) (Figure 6b). The certainty of evidence was rated as moderate.

**Figure 7:**
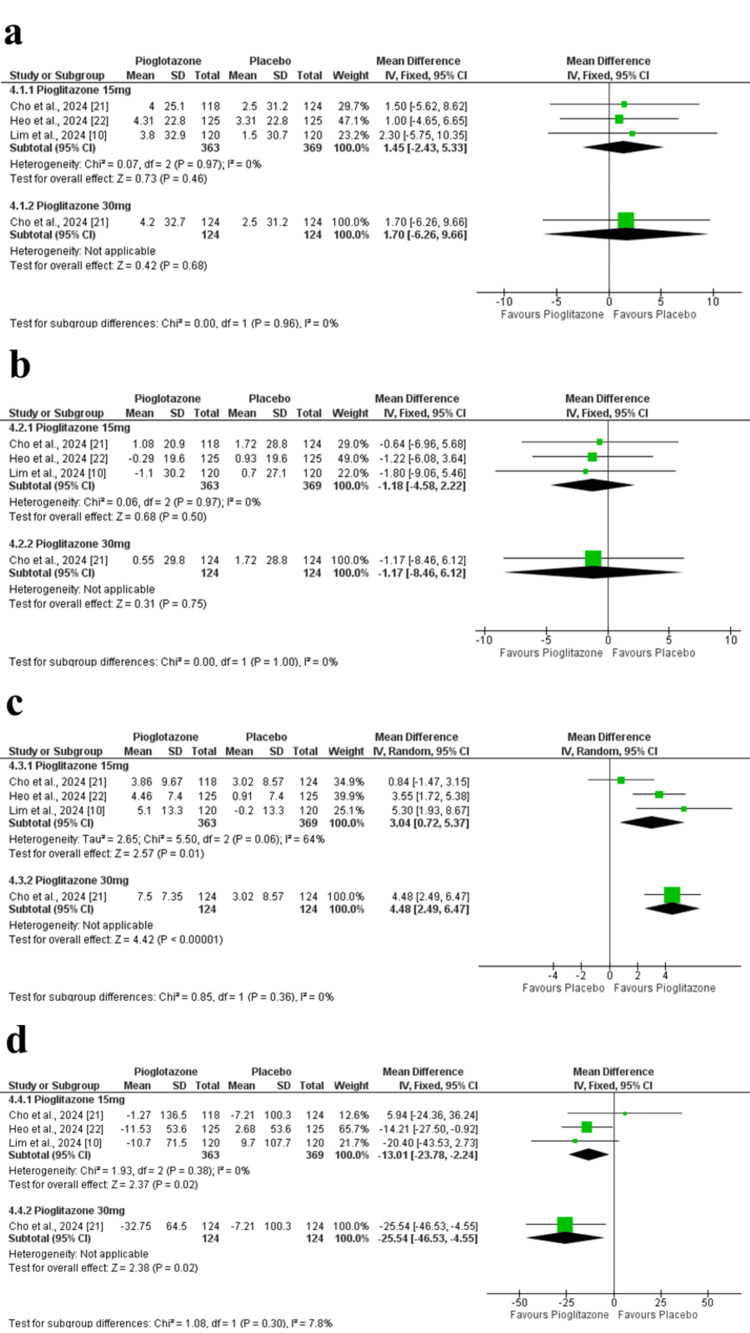
Forest plots of lipid profile outcomes at 24 weeks. (a) Change in total cholesterol. (b) Change in LDL-C. (c) Change in HDL-C. (d) Change in triglycerides. LDL-C: low-density lipoprotein cholesterol; HDL-C: high-density lipoprotein cholesterol

Total cholesterol: No significant differences were observed in total cholesterol between groups (MD = 1.45 mg/dl; 95% CI = -2.43 to 5.33; p = 0.63; I² = 0%). Similar findings were noted with the 30 mg dose in Cho et al. (2024) (MD = 1.70 mg/dL; 95% CI = -6.26 to 9.66; p = 0.68) (Figure 7a).

Low-density lipoprotein cholesterol: LDL-C levels did not significantly differ between groups (MD = -1.18 mg/dL; 95% CI = -4.58 to 2.22; p = 0.50; I² = 0%). The 30 mg dose also showed no effect (MD = -1.17 mg/dL; 95% CI = -8.46 to 6.12; p = 0.75) (Figure 7b). The certainty of evidence was rated as moderate.

High-density lipoprotein cholesterol: HDL-C significantly increased with pioglitazone 15 mg (MD = 3.04 mg/dL; 95% CI = 0.72 to 5.37; p = 0.01; I² = 64%). After excluding Cho et al. (2024) in a sensitivity analysis, heterogeneity decreased, and the result remained significant. The 30 mg dose further increased HDL-C (MD = 4.48 mg/dL; 95% CI = 2.49 to 6.47; p < 0.00001) (Figure 7c). The certainty of evidence was rated as moderate.

Triglycerides: Pioglitazone 15 mg significantly reduced triglycerides compared to placebo (MD = -13.01 mg/dL; 95% CI = -23.78 to -2.24; p = 0.03; I² = 0%). A greater reduction was seen with the 30 mg dose (MD = -25.54 mg/dL; 95% CI = -46.53 to -4.55; p = 0.02) (Figure 7d). The certainty of evidence was rated as moderate.

**Figure 8:**
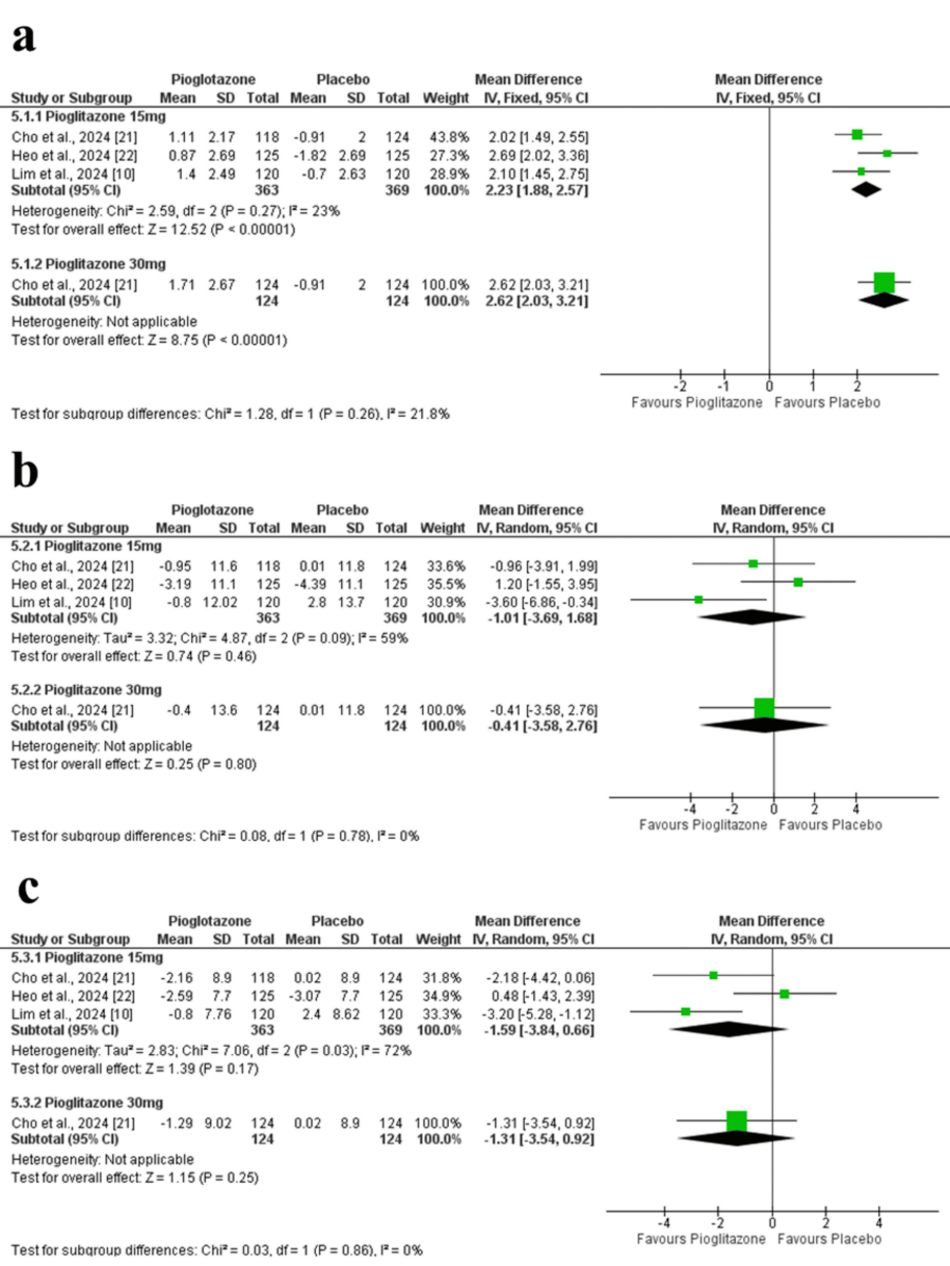
Forest plots of additional clinical outcomes at 24 weeks. (a) Change in body weight. (b) Change in SBP. (c) Change in DBP. SBP: systolic blood pressure; DBP: diastolic blood pressure

Body weight: Body weight increased significantly in the pioglitazone 15 mg group compared to placebo (MD = 2.23 kg; 95% CI = 1.88 to 2.57; p < 0.00001; I² = 23%). The 30 mg dose in Cho et al. (2024) showed a similar trend (MD = 2.62 kg; 95% CI = 2.03 to 3.21; p < 0.00001) (Figure 8a). The certainty of evidence was rated as high.

Systolic blood pressure: No significant change in SBP was observed (MD = -1.01 mmHg; 95% CI = -3.69 to 1.68; p = 0.46; I² = 59%). Sensitivity analysis excluding Lim et al. (2024) reduced heterogeneity. The 30 mg dose in Cho et al. (2024) also showed no effect (MD = -0.41 mmHg; 95% CI = -3.58 to 2.76; p = 0.80) (Figure 8b). The certainty of evidence was rated as low.

Diastolic blood pressure: DBP change was not significant (MD = -1.59 mmHg; 95% CI = -3.84 to 0.66; p = 0.17; I² = 72%). Excluding Heo et al. (2024) in the sensitivity analysis reduced heterogeneity. No difference was observed with the 30 mg dose (MD = -1.31 mmHg; 95% CI = -3.54 to 0.92; p = 0.25) (Figure 8c). The certainty of evidence was rated as low.

Safety Outcomes

**Figure 9:**
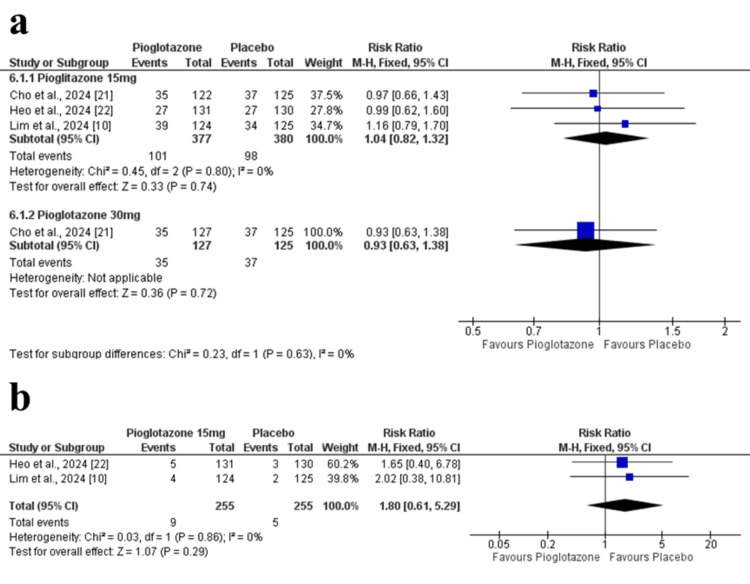
Forest plots of safety outcomes at 24 weeks. (a) Incidence of TEAEs. (b) Incidence of SAEs. TEAE: treatment-emergent adverse events; SAE: serious adverse event

Treatment-emergent adverse events: No significant difference was found in treatment-emergent adverse events between the pioglitazone 15 mg group and placebo (RR = 1.04; 95% CI = 0.82 to 1.32; p = 0.74; I² = 0%). The 30 mg dose also showed no difference (RR = 0.93; 95% CI = 0.63 to 1.38; p = 0.72) (Figure 9a). The certainty of evidence was rated as moderate.

Serious adverse events: Based on two studies, no significant difference was observed in SAEs between pioglitazone 15 mg and placebo (RR = 1.80; 95% CI = 0.61 to 5.29; p = 0.29; I² = 0%) (Figure 9b). The certainty of evidence was rated as moderate.

Discussion

This meta-analysis is the first to synthesize evidence on the efficacy and safety of pioglitazone as an add-on to dual therapy with metformin and dapagliflozin in adults with T2DM inadequately controlled on this combination. Across three RCTs involving 885 participants, pioglitazone significantly improved glycemic outcomes compared to placebo. The pooled results demonstrated meaningful reductions in HbA1c and FPG, with a more pronounced effect observed with the higher 30 mg dose evaluated in Cho et al. (2024). Triple therapy with pioglitazone was also associated with significant improvements in insulin resistance, as indicated by reductions in HOMA-IR. Although changes in HOMA-β were not statistically significant, the direction of effect suggests potential preservation of β-cell function. Favorable changes in lipid parameters were observed, including increased HDL-C and decreased triglycerides, while LDL-C and total cholesterol remained unchanged. SBP and DBP were also unaffected, indicating a neutral impact on blood pressure. The most consistent adverse effect was weight gain, observed with both 15 mg and 30 mg doses. However, adverse events did not differ significantly between the pioglitazone and placebo groups.

The addition of pioglitazone significantly reduced HbA1c levels at both 12 and 24 weeks. At 24 weeks, the pooled MD was -0.42% (95% CI = -0.51 to -0.33), and the 30 mg dose achieved a more pronounced reduction of -0.84% (95% CI = -1.02 to -0.66). These results are consistent with the individual RCTs: Lim et al. reported a -0.42% difference for the 15 mg dose​ and Cho et al. observed reductions of -0.38% and -0.83% for the 15 mg and 30 mg doses, respectively. Heo et al. also found a similar HbA1c reduction (-0.47%) with pioglitazone​ [10,21,22]. The consistency across trials underscores the robustness of the evidence. Additionally, a higher proportion of patients achieved glycemic targets with pioglitazone. Compared to placebo, the RR of achieving HbA1c <7% was 2.08 (95% CI = 1.65 to 2.62), and for <6.5%, the RR was 2.46 (95% CI = 1.54 to 3.93). These proportions were even more favorable at the 30 mg dose, with 52.4% and 18.6% of patients achieving <7% and <6.5%, respectively, in Cho et al. (2024) [21]. This suggests a dose-response relationship and supports the clinical utility of escalating to 30 mg where necessary.

Pioglitazone also significantly lowered FPG levels at both 12 and 24 weeks. At 24 weeks, the pooled MD was -12.41 mg/dL, and the 30 mg dose further reduced FPG by -21.49 mg/dL. Similar effects were reported in Heo et al. (-13.57 mg/dL), Lim et al. (-11.3 mg/dL), and Cho et al. (-11.71 mg/dL and -21.67 mg/dL for 15 and 30 mg doses, respectively) [10,21,22]. These consistent reductions reinforce the efficacy of pioglitazone in improving fasting glycemia across different trial settings. In previous trials using dipeptidyl peptidase-4 (DPP-4) inhibitors as add-on therapy with SGLT2 inhibitors and metformin, the proportion of patients achieving HbA1c <7% was much lower than with pioglitazone add-on therapy in our meta-analysis [23,24]. However, no prior meta-analysis has specifically examined the role of pioglitazone in triple therapy, underscoring the novelty of the present work. The significant reduction in HbA1c and FPG suggests that pioglitazone is a viable third-line therapy, potentially more effective than DPP-4 inhibitors in triple therapy.

We observed a significant improvement in HOMA-IR with pioglitazone 15 mg (MD = -0.79), while the 30 mg dose did not reach significance. These findings are in line with Heo et al., who reported a reduction of -0.78 (p < 0.0001) [22]. However, HOMA-β showed no significant change, suggesting that the main metabolic impact of pioglitazone in this regimen may be through enhancing insulin sensitivity rather than β-cell preservation. These effects are likely due to pioglitazone’s ability to enhance insulin sensitivity by activating PPAR-γ, which improves insulin sensitivity in skeletal muscle, liver, and adipose tissue. This mechanism promotes glucose uptake in muscle while simultaneously suppressing hepatic gluconeogenesis, leading to lower fasting glucose levels, HbA1c levels, and reduced insulin resistance. Additionally, pioglitazone helps preserve pancreatic β-cell function through its anti-inflammatory and antioxidant properties, supporting long-term glucose regulation and more effective diabetes management [25-27].

The addition of pioglitazone significantly increased HDL-C (MD = 3.04 mg/dL) and reduced triglycerides (MD = -13.01 mg/dL), findings consistent with Heo et al. (HDL-C = +3.67 mg/dL; TG = -16.01 mg/dL)​ and Cho et al. (HDL-C = +4.48 mg/dL; TG = -25.54 mg/dL)​ [21,22]. No significant changes were seen in LDL-C or total cholesterol. The effects on lipid metabolism are primarily mediated through the activation of PPAR-γ, which influences lipid metabolism, fat distribution, and insulin sensitivity. These metabolic benefits are congruent with known effects of pioglitazone and further support its use in patients with dyslipidemia. The certainty of evidence was rated moderate due to some imprecision and heterogeneity in HDL-C analysis.

A consistent increase in body weight was observed with pioglitazone (MD = +2.23 kg), which was expected given its fluid-retentive effect [28]. This aligns with the placebo-adjusted weight gain of 2.05-2.86 kg reported in Lim et al. and Heo et al. [10,22]​​. Edema was the most frequently reported adverse event with pioglitazone; however, its incidence was lower compared to previously reported trials [29]. This might be due to the addition of dapagliflozin, which helps in reducing fluid retention [30]. This is supported by the lower incidence of edema observed in Heo et al. [22] and Cho et al. [21]. Moreover, no significant effects on SBP or DBP were found across the studies, and heterogeneity was moderate to high for DBP. These findings suggest that while weight gain is a trade-off, it is not accompanied by adverse hemodynamic effects.

Adverse events did not significantly differ between pioglitazone and placebo, with pooled RRs close to 1. Cho et al. and Heo et al. both reported no major hypoglycemic events and comparable rates of adverse events (~20%) between groups. GRADE assessment rated the certainty of evidence for safety outcomes as moderate due to imprecision and low event rates. The 30 mg dose showed greater efficacy than the 15 mg dose without a significant increase in adverse events. These results support the use of pioglitazone in individualized treatment plans, particularly for patients with insulin resistance, while monitoring for weight changes and fluid retention. Notably, the most recent American Diabetes Association/European Association for the Study of Diabetes consensus has emphasized sequencing of therapies and combination strategies, which further supports the rationale for evaluating triple oral therapy regimens in contemporary practice [5]. In Lim et al. [10], two patients experienced hypoglycemic episodes in the pioglitazone group, but there were no major hypoglycemic events reported across the studies or drug-related SAEs. In Heo et al. [22], five SAEs were reported in the pioglitazone group (diverticulitis, otitis media, pneumonia, intervertebral disc protrusion, and diabetic foot), while in Lim et al. [10], an ankle fracture was reported, but none of these events were directly linked to pioglitazone. Earlier observational studies had raised concerns regarding a possible association between pioglitazone and bladder cancer; however, large cohort analyses such as Lewis et al. (2015) suggest that the absolute risk is low and not clinically significant [31]. Furthermore, consistent with findings from the PROactive trial, clinicians should remain vigilant about the potential for fluid retention and increased risk of heart failure with pioglitazone, particularly in patients with pre-existing cardiac disease [9].

Additionally, the IRIS trial offered more long-term safety and effectiveness data, showing that pioglitazone decreased the risk of major vascular events and recurrent stroke in patients with insulin resistance but not diabetes. These results are consistent with the outcomes of our included studies and support the wider cardiovascular safety profile of pioglitazone, despite being conducted in a different population [32].

Compared to other triple therapies, the main limitation of the combination of pioglitazone as an add-on to metformin and dapagliflozin would be its weight gain side effect [33]. In contrast, triple combinations utilizing glucagon-like peptide-1 receptor agonists (GLP-1 RAs) as an add-on therapy to metformin and dapagliflozin promote significant weight loss, which would make this combination a better-suited option for patients with comorbid obesity, metabolic syndrome, and high cardiovascular risk [34]. In addition, GLP-1 RAs have proven cardiovascular and renal protective effects, which pioglitazone might lack. This could make them a more favorable option, especially for patients with chronic kidney disease [35].

Strengths

Our study has several strengths. To our knowledge, it is the first meta-analysis evaluating the efficacy and safety of pioglitazone added to metformin and dapagliflozin. All included studies were high-quality RCTs with well-matched baseline characteristics and consistent interventions. By focusing specifically on the triple therapy of metformin, dapagliflozin, and pioglitazone, we address a clinically relevant question with direct implications for patient care. Additionally, all included studies were double-blind, multicenter RCTs. We also assessed both 15 mg and 30 mg pioglitazone doses, the latter showing greater efficacy in reducing HbA1c. Finally, we used the GRADE assessment to classify the certainty of the evidence, which showed high certainty for all primary outcomes.

Limitations

This review has certain limitations. Only three RCTs with short follow-up (24 weeks) were included, all conducted in Korea, which restricts generalizability. The background metformin dose (1,000 mg/day) was lower than in routine practice (up to 2,000 mg/day). Safety analyses were underpowered for rare but clinically important events, and publication bias could not be formally assessed. For some outcomes, substantial heterogeneity was observed, but its sources could not be identified. Finally, although we performed subgroup analyses by pioglitazone dose (15 mg vs. 30 mg), meta-regression to assess dose-response was not feasible due to the limited number of trials.

## Conclusions

This meta-analysis demonstrates that adding pioglitazone to metformin and dapagliflozin significantly improves glycemic control, insulin sensitivity, and lipid parameters with an acceptable safety profile in adults with T2DM inadequately controlled on dual therapy. These findings suggest that pioglitazone may be a valuable third-line option in carefully selected patients, but larger and longer-term studies are needed to confirm the durability of benefits and clarify long-term safety.

## Appendices

 Appendix 1

**Table 4:** Search strategy for each database.

Website	Search strategy	Filter	Number
PubMed	(Pioglitazone OR AD4833 OR U72107A OR Pioglitazone Hydrochloride OR Actos) AND (Dapagliflozin OR BMS512148 OR Farxiga OR Forxiga) AND (Metformin OR Dimethylbiguanidine OR Glucophage OR Dimethylguanylguanidine OR Metformin Hydrochloride) AND (Type 2 diabetes OR Type 2 DM)	No filter	31
Cochrane	(Pioglitazone OR AD4833 OR U72107A OR Pioglitazone Hydrochloride OR Actos) AND (Dapagliflozin OR BMS512148 OR Farxiga OR Forxiga) AND (Metformin OR Dimethylbiguanidine OR Glucophage OR Dimethylguanylguanidine OR Metformin Hydrochloride) AND (Type 2 diabetes OR Type 2 DM)	No filter	42
Web of Science	(Pioglitazone OR AD4833 OR U72107A OR Pioglitazone Hydrochloride OR Actos) AND (Dapagliflozin OR BMS512148 OR Farxiga OR Forxiga) AND (Metformin OR Dimethylbiguanidine OR Glucophage OR Dimethylguanylguanidine OR Metformin Hydrochloride) AND (Type 2 diabetes OR Type 2 DM)	No filter	67
Scopus	(“pioglitazone” AND “dapagliflozin” AND “metformin” AND “type 2 diabetes”)	Limited to Medicine, article, English	200

 Appendix 2

**Table 5:** Detailed grading of evidence and recommendations (GRADE). HbA1c: hemoglobin A1c; FPG: fasting plasma glucose; HOMA-IR: homeostatic model assessment of insulin resistance; HOMA-B: homeostatic model assessment of beta-cell function; HDL: high-density lipoprotein; LDL: low-density lipoprotein; SBP: systolic blood pressure; DBP: diastolic blood pressure; RR: relative risk; MD: mean difference; CI: confidence interval Explanations: a wide confidence interval.

Certainty assessment							Number of patients		Effect		Certainty
Number of studies	Study design	Risk of bias	Inconsistency	Indirectness	Imprecision	Other considerations	Piogletazone	placebo	Relative (95% CI)	Absolute (95% CI)	
HbA1C - (follow-up: range 1 week to 24 weeks)											
3	Randomized trials	Not serious^a^	Not serious	Not serious	Not serious	None	363	369	-	MD 0.42 % lower (0.51 lower to 0.33 lower)	⨁⨁⨁⨁ High^a^
HbA1c - (follow-up: range 1 week to 12 weeks)											
3	Randomized trials	Not serious	Not serious	Not serious	Not serious	None	363	369	-	MD 0.33 % lower (0.41 lower to 0.25 lower)	⨁⨁⨁⨁ High
HbA1C < 7% - (follow-up: range 1 week to 24 weeks)											
3	Randomized trials	Not serious	Not serious	Not serious	Not serious	None	153/357 (42.9%)	74/361 (20.5%)	RR 2.08 (1.65 to 2.62)	23 more per 100 (from 14 more to 33 more)	⨁⨁⨁⨁ High
HbA1C < 6.5% - (follow-up: range 1 week to 24 weeks)											
3	Randomized trials	Not serious	Not serious	Not serious	Not serious	None	54/357 (15.1%)	22/361 (6.1%)	RR 2.46 (1.54 to 3.93)	9 more per 100 (from 3 more to 18 more)	⨁⨁⨁⨁ High
FPG - (follow-up: range 1 week to 24)											
3	Randomized trials	Not serious	Not serious	Not serious	Not serious	None	363	369	-	MD 12.41 mg/dL lower (15.66 lower to 9.15 lower)	⨁⨁⨁⨁ High
FPG - (follow-up: range 1 week to 12 weeks)											
3	Randomized trials	Not serious	Not serious	Not serious	Not serious	None	363	369	-	MD 10.6 % lower (14.11 lower to 7.1 lower)	⨁⨁⨁⨁ High
HOMA-IR - (follow-up: range 1 week to 24 weeks)											
3	Randomized trials	Not serious	Not serious	Not serious	Not serious	None	363	369	-	MD 0.79 % lower (1.06 lower to 0.53 lower)	⨁⨁⨁⨁ High
HOMA-B - (follow-up: range 1 week to 24 weeks)											
2	Randomized trials	Not serious	Not serious	Not serious	Serious^a^	None	243	249	-	MD 1.08 % higher (5.84 lower to 8 higher)	⨁⨁⨁◯ Moderate^a^
Total cholesterol - (follow-up: range 1 week to 24 weeks)											
3	Randomized trials	Not serious	Not serious	Not serious	Serious^a^	None	363	369	-	MD 1.45 mg/dL higher (2.43 lower to 5.33 higher)	⨁⨁⨁◯ Moderate^a^
HDL - (follow-up: range 1 week to 24 weeks)											
3	Randomized trials	Not serious	Serious^a^	Not serious	Not serious	None	363	369	-	MD 2.93 mg/dL higher (1.61 higher to 4.25 higher)	⨁⨁⨁◯ Moderate^a^
LDL - (follow-up: range 1 week to 24 weeks)											
3	Randomized trials	Not serious	Not serious	Not serious	Serious^a^	None	363	369	-	MD 1.18 mg/dL lower (4.58 lower to 2.22 higher)	⨁⨁⨁◯ Moderate^a^
Triglyceride - (follow-up: range 1 week to 24 weeks)											
3	Randomized trials	Not serious	Not serious	Not serious	Serious^a^	None	363	369	-	MD 13.01 mg/dL lower (23.78 lower to 2.24 lower)	⨁⨁⨁◯ Moderate^a^
Body weight - (follow-up: range 1 week to 24 weeks)											
3	Randomized trials	Not serious	Not serious	Not serious	Not serious	None	363	369	-	MD 2.23 Kg higher (1.88 higher to 2.57 higher)	⨁⨁⨁⨁ High
SBP - (follow-up: range 1 week to 24 weeks)											
3	Randomized trials	Not serious	Serious^a^	Not serious	Serious^a^	None	363	369	-	MD 0.85 mmHg lower (2.56 lower to 0.86 higher)	⨁⨁◯◯ Low^a^
DBP - (follow-up: range 1 week to 24 weeks)											
3	Randomized trials	Not serious	Serious^a^	Not serious	Serious^a^	None	363	369	-	MD 1.48 mmHg lower (2.67 lower to 0.29 lower)	⨁⨁◯◯ Low^a^
Treatment-emergent adverse events - (follow-up: range 1 week to 24 weeks)											
3	Randomized trials	Not serious	Not serious	Not serious	Serious^a^	None	101/377 (26.8%)	98/380 (25.8%)	RR 1.04 (0.82 to 1.32)	1 more per 100 (from 5 fewer to 8 more)	⨁⨁⨁◯ Moderate^a^
serious adverse events - (follow-up: range 1 week to 24 weeks)											
2	Randomized trials	Not serious	Not serious	Not serious	Serious^a^	None	9/255 (3.5%)	5/255 (2.0%)	RR 1.8 (0.61 to 5.29)	2 more per 100 (from 1 fewer to 9 more)	⨁⨁⨁◯ Moderate^a^

Appendix 3 

**Table 6:** Risk of bias in randomization process. D: domain; Y: yes; PY: probably yes; N: no; NI: no information; SAS: statistical analysis system; HbA1c: hemoglobin A1c

Studies	D1: Randomization process							
	Sequence generation		Allocation concealment		Baseline balance differences		Final decision	
	Authors’ judgment	Comment	Authors’ judgment	Comment	Authors’ judgment	Comment	Authors’ judgment	Comment
Heo et al. (2024) [22]	NI	No mentioned method	NI	No mentioned method	N	Patient demographics and baseline characteristics were balanced between the two treatment groups in the FAS	Some concern	-
Lim et al. (2024) [10]	Y	Randomization was conducted using an interactive web response system and stratified by baseline HbA1c level (<8.0% or ≥8.0%)	PY		N	There were no significant differences in baseline characteristics between the two groups	Low	-
Cho et al. (2024) [21]	Y	Stratified block randomization was executed at each site using SAS software version 9.4 (SAS Institute, Cary, North Carolina) within the framework of a double-blind study protocol	PY	Participants eligible for the study were randomly allocated	N	Baseline demographic and clinical characteristics were well-matched among the four groups analyzed based on the efficacy data	Low	-

Appendix 4 

**Table 7:** Risk of bias in deviations from intended interventions. D: domain; Y: yes; N: no; ITT: intention-to-treat; HbA1c: hemoglobin A1c

Studies	D2: Deviations from intended interventions															
	Participants aware		Investigators awareness		Were there deviations arose because of the experimental context		Deviations affected the outcome		Balance between groups		Appropriate analysis		Potential impact of the failure to analyse participants		Final decision	
	Authors’ judgment	Comment	Authors’ judgment	Comment	Authors’ judgment	Comment	Authors’ judgment	Comment	Authors’ judgment	Comment	Authors’ judgment	Comment	Authors’ judgment	Comment	Authors’ judgment	Comment
Heo et al. (2024) [22]	N	Blinding was performed	N	Blinding was performed	N		N		Y		Y	The full analysis set consisted of patients who were exposed to at least one dose of the study drug and had at least one HbA1c result after randomization	N	The full analysis set consisted of patients who were exposed to at least one dose of the study drug and had at least one HbA1c result after randomization	Low	-
Lim et al. (2024) [10]	N	Double-blind	N	Double-blind	N		N		Y		Y	The ITT set was analyzed as the main analysis group	N	The ITT set was analyzed as the main analysis group	Low	-
Cho et al. (2024) [21]	N	Double-blind	N	Double-blind	N		N		Y		Y	Overall analysis was performed on the full analysis set	N	Overall analysis was performed on the full analysis set	Low	-

 Appendix 5

**Table 8:** Risk of bias in measurement of the outcome. D: domain; PN: probably no; N: no

Studies	D3: Bias in measurement of the outcome									
	Method of measuring the outcome inappropriate.		Differed between intervention groups.		Were outcome assessors aware?		Assessment of the outcome have been influenced by knowledge.		Final decision	
	Authors’ judgment	Comment	Authors’ judgment	Comment	Authors’ judgment	Comment	Authors’ judgment	Comment	Authors’ judgment	Comment
Heo et al. (2024) [22]	N	-	N	-	PN	-	PN	-	Low	-
Lim et al. (2024) [10]	N	-	N	-	PN	-	PN	-	Low	-
Cho et al. (2024) [21]	N	-	N	-	PN	-	N	-	Low	-

Appendix 6 

**Table 9:** Risk of bias in missing outcome data. D: domain; Y: yes; N: no

Studies	D4: Bias due to missing outcome data									
	Outcome data for all participants?		Evidence that the result is not biased		Messiness could depend on the true value		Messiness is likely to depend on the true value		Final decision	
	Authors’ judgment	Comment	Authors’ judgment	Comment	Authors’ judgment	Comment	Authors’ judgment	Comment	Authors’ judgment	Comment
Heo et al. (2024) [22]	N	There is missing data	Y	Presence of a full analysis set	N	-	N	-	Low	-
Lim et al. (2024) [10]	N	Presence of missing outcome data and withdrawal from the study	Y	Presence of Intention-to-treat analysis	N	-	N	-	Low	-
Cho et al. (2024) [21]	N	Presence of missing outcome data and withdrawal from the study	Y	Presence of full analysis set	N	-	N	-	Low	-

 Appendix 7

**Table 10:** Risk of bias in selection of the reported result. D: domain; Y: yes; PY: probably yes; N: no; SAS: statistical analysis system

Studies	D5: Bias in selection of the reported result					
	Results selected from multiple measurements or analyses		Trial analysed in accordance		Final decision	
	Authors’ judgment	Comment	Authors’ judgment	Comment	Authors’ judgment	Comment
Heo et al. (2024) [22]	N	All statistical analyses were prespecified	Y	All statistical analyses were performed using SAS version. Section 9.4 (SAS Institute, Cary, NC, USA)	Low	-
Lim et al. (2024) [10]	N	All statistical analyses were prespecified.	PY		Low	-
Cho et al. (2024) [21]	N	All statistical analyses were prespecified	Y	Statistical analyses were performed using SAS software version 9.4	Low	-

Appendix 8 

**Table 11:** Other risk of bias. N: no

Studies	Other bias			
					Authors’ judgment	Comment	Authors’ judgment	Comment
									Heo et al. (2024) [22]	N	-	Low	-
	Lim et al. (2024) [10]	N	-	Low					-
	Cho et al. (2024) [21]	N	-	Low					-
